# Isotopic graphene–isolated-Au-nanocrystals with cellular Raman-silent signals for cancer cell pattern recognition†
†Electronic supplementary information (ESI) available. See DOI: 10.1039/c7sc05442d


**DOI:** 10.1039/c7sc05442d

**Published:** 2018-02-12

**Authors:** Yuxiu Zou, Siqi Huang, Yixin Liao, Xupeng Zhu, Yiqin Chen, Long Chen, Fang Liu, Xiaoxiao Hu, Haijun Tu, Liang Zhang, Zhangkun Liu, Zhuo Chen, Weihong Tan

**Affiliations:** a Molecular Science and Biomedicine Laboratory (MBL) , State Key Laboratory of Chemo/Bio-Sensing and Chemometrics , College of Chemistry and Chemical Engineering and College of Life Sciences , Aptamer Engineering Center of Hunan Province , Hunan University , Changsha , Hunan 410082 , China . Email: zhuochen@hnu.edu.cn; b State Key Laboratory of Advanced Design and Manufacturing for Vehicle Body , College of Mechanical and Vehicle Engineering , Hunan University , Changsha , Hunan 410082 , China; c Faculty of Science and Technology , University of Macau , E11, Avenida da Universidade , Taipa , 999078 , Macau; d Department of Chemistry and Department of Physiology and Functional Genomics , Center for Research at Bio/nano Interface , Health Cancer Center , UF Genetics Institute and McKnight Brain Institute , University of Florida , Gainesville , Florida 32611-7200 , USA

## Abstract

For cancer diagnosis, technologies must be capable of molecular recognition, and they must possess a built-in pattern recognition component for efficient imaging and discrimination of targeted cancer cells. Surface enhanced Raman scattering (SERS) tags based on plasmonically active nanoparticles hold promise for accurate and efficient cancer cell recognition, owing to ultra-narrow peak and sensitive optical properties. However, a complex fingerprint spectrum increases data analysis difficulty, making it necessary to develop multicolor SERS tags with a simple fingerprint spectrum. To address this, we herein fabricated SERS-encoded nanoparticles (NPs) with stable and simple fingerprint spectrum through synthesis of isotopic cellular Raman-silent graphene–isolated-Au-nanocrystals (GIANs) and conjugation with phospholipid-polyethylene glycol-linked aptamers to target proteins overexpressed on the cancer cell surface. GIANs, which possess the properties of graphitic nanomaterials, such as super-stable optical properties and high Raman cross-section, showed enhanced SERS signals. The 2D-band Raman shift of GIAN, which located in the cellular Raman-silent region, was easily regulated through fabrication of isotopic GIANs without changing their molecular structure. Such GIAN tags demonstrated multiplexed Raman imaging capability, both *in vivo* and *in vitro*, with low background interference. Moreover, cell membrane protein (nucleolin, mucin and epithelial cell adhesion molecule)-specific, aptamer-conjugated isotopic GIANs were fabricated and feasibly applied to built-in coding for rapid imaging and pattern recognition of targeted cancer cells. Such isotopic GIAN-aptamer-encoders show high potential for efficient cancer cell identification and diagnosis.

## Abstract 2

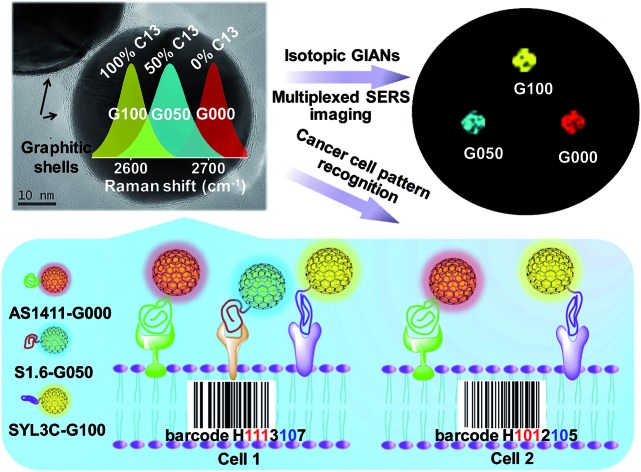
Isotopic graphene–isolated-Au-nanocrystal SERS tags with simple, low background and super-stable fingerprint spectra were developed for pattern recognition of cancer cells.

## Introduction

Multiplexed, sensitive and specific molecular detection is highly desirable for environmental analysis, drug screening, gene and protein profiling, as well as clinical diagnostics (*e.g.*, cancer).^1^–^4^ Simultaneous detection of multiple tumor-related proteins on the cell surface would prove invaluable for accurate cancer diagnostics and help in understanding the roles of these proteins in cancer development. To perform such multiplexed detection, one strategy would utilize bioconjugated “encoders” to identify the attached ligand molecules.^5^–^8^ In fact, optical encoding methods, such as fluorescence, SERS, reflection, and luminescence lifetime, are attracting more attention for their rapid application.^9^–^16^ However, reflection- and luminescence lifetime-encoding strategies involve relatively complicated encoding processes. Organic dye-based encoders suffer from several drawbacks, as well.^17^ For instance, multiple excitation lasers are required when dyes with different excitation wavelengths are used, which is costly.^12^ Moreover, interference between fluorescence encoders is inevitable, making it necessary to compensate by using complicated and tedious color arrangements. Owing to narrow and tunable emissions excited at the same wavelength, quantum dots (QDs) could overcome the limitations of organic dyes.^14^ However, the biological application of QDs was limited by their complex structure, surface chemistry and cytotoxicity.^18^,^19^ When developing optical-encoded NPs for bioimaging, the cytotoxicity, size, and long-term colloidal stability of NPS, as well as the stability of optical properties, must be considered.^20^ Recently, SERS technologies,^21^–^28^ which possess the merits of QDs, have developed rather quickly as a result of the high sensitivity, anti-photobleaching and ultra-narrow Raman peak.^29^ The peak width of Raman bands is approximately 1–2 nm, about 100–10 times narrower than that of fluorescence emission from organic dyes or QDs.^30^,^31^ Encoded SERS NPs seem promising for sensitive multiplexed detection.^32^–^34^ In cancer diagnostics, multiplexed molecular recognition is required, but still insufficient by the need to discriminate among cancer cell types. Thus, a built-in pattern recognition component for efficient imaging and typing of targeted cancer cells is also required.^35^,^36^ However, conventional SERS tags showed low photostability under long irradiation time and complex fingerprint spectra, making data analysis increasingly difficult. Thus, novel multiplexed SERS tags integrated high stability and simple fingerprint spectral are desired. Herein, we report the fabrication of biocompatible multicolor SERS tags based on regulating Raman shift of intact graphene coated on AuNPs by the chemical vapor deposition (CVD) method, defined as graphene-isolated–Au-nanocrystals, or GIANs. Intact graphene was deposited on AuNPs, providing a protective shell. Owing to this graphitic shell, GIAN tags were ultra-stable and resistant to oxidation with a full range of pH and capacity for powerful laser irradiation.^37^–^39^ Importantly, the graphitic shell of GIANs showed unique and simple Raman signal, a disordered D-band (1355 cm^–1^), a graphitic carbon G-band (1590 cm^–1^) and a 2D-band (2706 cm^–1^) at about twice the frequency of the D-band and utilized for sensitive SERS imaging.^38^,^40^ The high intensity (relative to the G peak) Raman D peak reflects the high strain of the graphitic shells as a result of the deformation of the flattened graphene encapsulating the Au nanocrystals. It is notable that the graphitic shell provided super-stable optical properties and strong 2D-band located in the cellular Raman-silent region free from interference of biomolecules (1800–2800 cm^–1^), making it possible to realize precision bioimaging.^39^,^41^ Moreover, multiplexed GIAN SERS tags with Raman signals located in the Raman-silent region could be designed and synthesized, and the 2D-band of GIANs could be regulated through the introduction of isotopic carbon compositions.^42^ Varying the fraction of isotopic compositions of C12 and C13, five GIAN multiplexed tags were synthesized. Compared to traditional SERS tags, these unique isotopic GIAN multicolored tags were conferred with low interference from background, super-stable SERS signals, well-regulated Raman shift and facile bioconjugation.^15^,^34^,^43^,^44^ Instead of regulating Raman shift through the design of spectroscopic derivatives,^44^ all atoms of a molecule were replaced with stable isotopes of the same element such that the molecule's chemical structure and intensity of vibrational bands remained nearly unchanged. However, the vibrational frequencies of these bands did have distinct changes.^45^ Multiplexed *in vivo* and *in vitro* Raman imaging with low background interference was demonstrated with such isotopic GIAN SERS tags.

To use a GIAN-encoder for cancer cell identification, a cell-specific ligand is necessary. Aptamers are oligonucleic acid molecules that have specificity and affinity to a wide range of targets that vary from small molecules to cancer cells.^46^,^47^ In contrast to antibodies, aptamers are cost-effective chemical antibodies that can be chemically modified and synthesized using an automatic DNA synthesizer, ensuring reproducibility from batch to batch. Many aptamers have been selected by cell-SELEX methods.^48^–^50^ Here, three specific phospholipid-polyethylene glycol-linked aptamers, including AS1411 targeting nucleolin,^51^ S1.6 binding mucin protein (MUC1) and SYL3C targeting epithelial cell adhesion molecule (EpCAM), were synthesized for conjugation with the isotopic GIAN-encoders through hydrophobic–hydrophobic interaction, respectively.^52^–^54^ The aptamer-conjugated isotopic GIAN-encoders incorporated a built-in pattern recognition component for simultaneous, rapid and targeted cancer cell imaging. According to the different SERS barcodes, two cancer cell lines were easily distinguished. Such unique GIAN-encoders showed high promise as a diagnostic tool for efficient cancer cell targeting and discrimination.

## Results and discussion

### Synthesis and characterization of GIANs

**Fig. 1:**
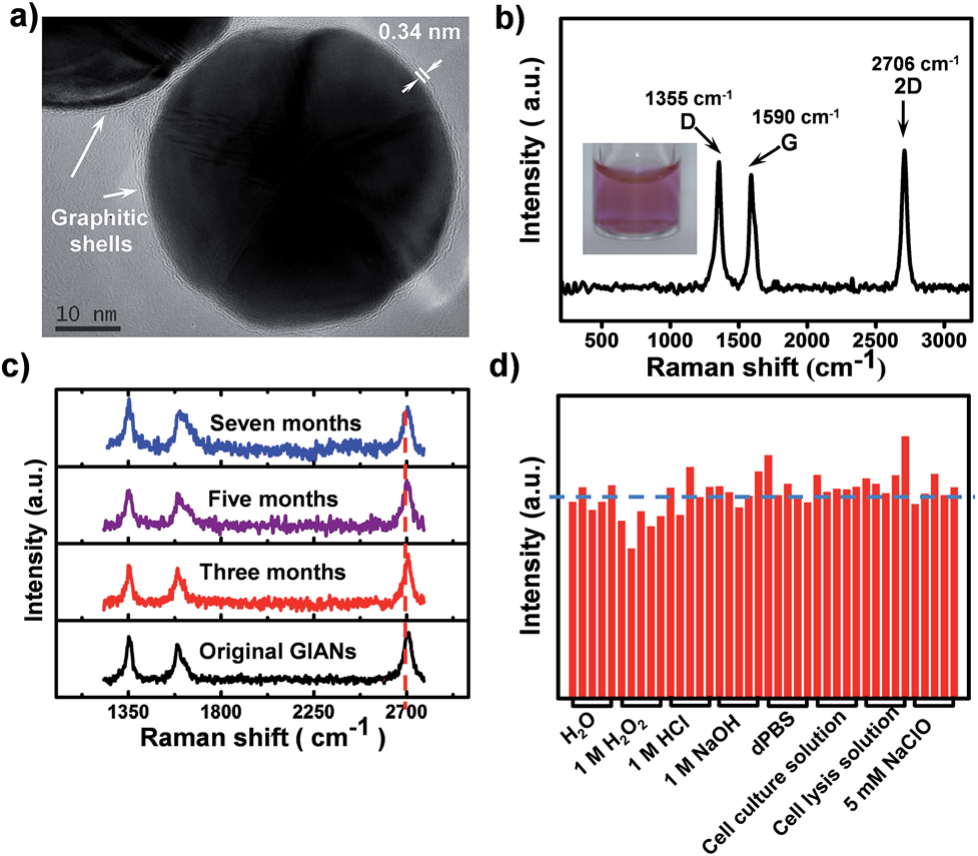
Advanced characterization of GIANs. (a) HR-TEM image of GIANs. (b) SERS spectrum of aqueous GIANs suspension. Inset in (b) presented the image of aqueous GIAN suspension. (c) SERS spectra of GIANs stored in water for various time. (d) SERS intensity stability of 2D-band of GIANs after incubating with H_2_O, 1 M H_2_O_2_, 1 M HCl, 1 M NaOH, Dulbecco's phosphate buffered solution (dPBS), cell culture solution, cell lysis solution, and 5 mM NaClO for 3 hours.

GIANs were made by the CVD method.^55^ High-resolution transmission electron microscopy (HR-TEM) was utilized to characterize the GIAN nanocrystal (Fig. 1a). A few layer graphene, less than 5 layers was deposited intact on AuNPs as a protective shell. The space between shell layers was ∼0.34 nm, which is consistent with the interlayer distance of graphite. The average size of GIANs was around 45 nm (Fig. S1a†). As shown in Fig. 1b, GIANs exhibited very simple and unique Raman spectrum, D-band (1355 cm^–1^), G-band (1590 cm^–1^) and 2D-band (2706 cm^–1^) under the 532 nm laser, representing the typical Raman spectrum of graphitic nanomaterial. High intensity of D-band was observed which might be due to the high strain of the graphitic shells, as well as the inducing of the detects during the GIAN synthesis. The graphitic shell provided a strong 2D-band located in the cellular Raman-silent region free from interference of biomolecules (1800–2800 cm^–1^). 633 nm laser was also used to excite GIANs. However, the SERS intensity of 2D band was decreased sharply. Thus 532 nm laser was selected for all the SERS analysis consequently. To achieve better solubility, a polyoxyethylenestearyl ether (C_18_-PEG) molecule was introduced to functionalize GIANs through hydrophobic–hydrophobic interaction. DLS (Fig. S2†) were used to characterize the functionalized process. Consequently, GIANs were adequately dispersed in water, and the GIAN solution demonstrated a transparent plum, as shown in Fig. 1b (inserted image).

Such GIAN nanoparticle allows facile surface functionalization and can be stored as either powder or solution. GIANs have two absorbance peaks in the UV-Vis curve (Fig. S1b†). The plasmonic resonance absorbance of GIANs located in 560 nm came from the gold nanocrystal core, while the peak at about 265 nm came from the graphitic shell of GIANs. The relationship between full width at half-maximum (FWHM) of Raman 2D-band intensity and UV-Vis absorbance ratios of peak 265 nm and 560 nm (*A*_265_/*A*_560_) was also investigated. By increasing CVD growth time, the ratio of *A*_265_/*A*_560_ increased, respectively. GIANs with narrowest Raman FWHM and strongest SERS signal were realized within 8 minutes (Fig. S1c and d†). As illustrated in Fig. 1c, aqueous GIANs suspension presented super-stable optical properties. The SERS spectra of GIANs aqueous solution showed no obvious changes, even after seven months of storage. Moreover, the SERS 2D-band intensity of GIANs remained super-stable, even when incubated with such harsh and complex solutions as H_2_O_2_, HCl, NaOH, NaClO or cell lysis solution (Fig. 1d). As shown in the images and UV-Vis spectra, GIANs presented superior stability, even after eight hours of incubation in harsh and complex solution (Fig. S3 and S4†). The detection sensitivity of GIANs solution was high (Fig. S5†), and the electric field distribution of 45 nm GIAN was simulated by the finite-difference time-domain (FDTD) simulation under different laser excitation (Fig. S6†).

### Multiplexed isotopic GIANs SERS tags

**Fig. 2:**
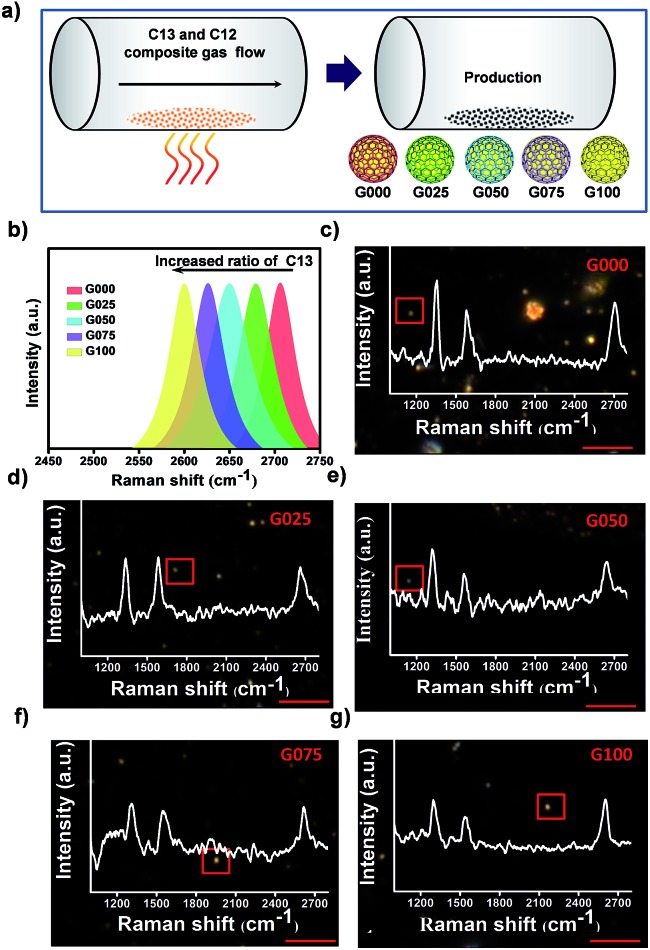
Raman characterization of multiplexed isotopic GIANs. (a) Schematic illustration of synthesis of multiplexed GIAN tags, defined as G100 (100% C13), G075 (75% C13), G050 (50% C13), G025 (25% C13), and G000 (0% C13). (b) SERS spectra of multiplexed GIANs suspension. Under the leading of dark-field imaging, SERS spectra on few (c) G000, (d) G025, (e) G050, (f) G075 and (g) G100 particles boxed in respect dark-field image; scale bar, 5 μm.

Through rational design of the CVD synthesis gas source, multiplexed GIAN tags were fabricated. The synthetic strategy for five unique GIAN tags was schematically illustrated in Fig. 2a. The details on the preparation of GIANs were given in ESI.† By introducing the isotopic carbon source, multiplexed isotopic GIAN tags were achieved by varying the compositions of C13 and C12 methane during the CVD process.^42^,^56^–^61^ GIANs grown with gas phase C13 composition ratios at 100%, 75%, 50%, 25%, and 0% exhibited 2D-band peaks at 2600 cm^–1^, 2625 cm^–1^, 2650 cm^–1^, 2680 cm^–1^, and 2706 cm^–1^, which were defined as G100, G075, G050, G025, and G000, respectively (Fig. 2b). Under the leading of dark-field microscopy imaging of few isotopic particles, the Raman shift of each few isotopic particles was obtained that consistent with bulk suspension. Optical scattering experiments on few isotopic GIANs demonstrated high sensitivity and precise Raman shift (Fig. 2c–g). Additionally, the whole synthesis procedure was simple and fast. The 2D-band peaks between GIANs were within the range of 2600 to 2706 cm^–1^, representing a shift of about 106 cm^–1^. Of note, we observed that the Raman shifts of 2D-band were 1.9 and 1.8 times greater than those of D- and G-band, respectively (Fig. S7†). These results indicated that the multiplexed tags with 2D-band showed spectrum resolution superior to that of multiplexed tags with D-band or G-band. The phenomenon of larger down shift of 2D band was discussed in ESI.†

### SERS imaging with cellular Raman-silent GIANs *in vitro* and *in vivo*

**Fig. 3:**
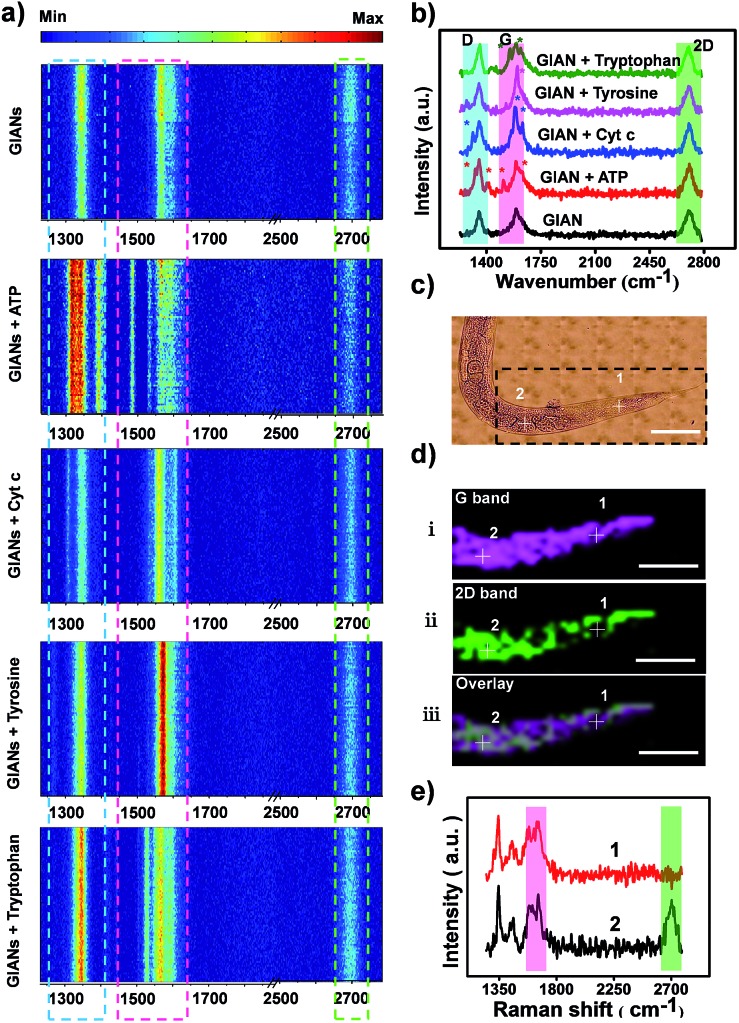
The anti-interference ability of GIANs *in vitro* and *in vivo*. (a) Maps of 81 SERS spectra on GIANs mixed with the biomolecules ATP, cytochrome c, tyrosine, tryptophan and the control, respectively. (b) SERS spectra of GIANs mixed with biomolecules corresponding to (a). (c) Bright-field of *C. elegans*. (d) SERS mapping images of *C. elegans* corresponding to boxed area in (c). (i) G-band, 1590 cm^–1^ (plum); (ii) 2D-band, 2706 cm^–1^ (green); (iii) overlay. Step size, 11 μm, scale bar, 200 μm. (e) SERS spectra marked in SERS mapping images of *C. elegans* (d).

The D-band and G-band of graphitic nanomaterials proved to be superior SERS tags for cancer cell imaging.^55^ However, similar to some organic SERS reporters, both D-band and G-band of GIANs could overlap with the Raman peaks derived from endogenous biomolecules in the cells. Overlapping signals are difficult to fully resolve, thus influencing detection accuracy. Research on the design and synthesis of multiplexed SERS tags without interference from biomolecules has been rare. Fortunately, the 2D-band of multiplexed isotopic GIAN tags located in the cellular Raman-silent region (1800–2800 cm^–1^) exhibited low background interference, thereby improving the accuracy of SERS imaging.^62^–^64^ Fig. 3a showed the maps of 81 SERS spectra of G000s mixed with the biomolecules adenosine triphosphate (ATP), cytochrome c (Cyt c), tyrosine, and tryptophan, respectively. As illustrated in these SERS maps, compared to pure G000s, the D- or G-band of G000s mixtures overlapped with the Raman peak of biomolecules, while the 2D-band region showed low background interference. The peaks of these biomolecules were marked in Fig. 3b. These results revealed the accurate Raman imaging potential of GIAN SERS tags.

We further demonstrated the merit of GIANs as SERS tags *in vivo* by using *C. elegans* as a model. To accomplish this, pseudocoel of *C. elegans* was microinjected with G000s. Spectroscopic mapping of *C. elegans* was then carried out. The G and 2D peak intensity of GIANs at each pixel was integrated to construct the SERS image. The pseudocoel of *C. elegans* boxed in Fig. 3c was stained by G-band (1590 cm^–1^, plumcolor) and 2D-band (2706 cm^–1^, green color) of G000s (Fig. 3d). In some cases, SERS bioimaging with 1590 cm^–1^ G-band did, inevitably, lead to background noise. Note that endogenous proteins can produce vibration at 1200–1650 cm^–1^, thus producing spectral overlap. As illustrated in Fig. 3d(i), pixel 1 was illuminated. However, according to the SERS spectrum of pixel 1 (Fig. 3e), no 2D-band was presented. The spectrum of pixel 1 showed a false-positive SERS signal of G-band that gave rise to the background Raman signal of *C. elegans*. In contrast, as illustrated in Fig. 3d(ii), pixel 2 was illuminated by GIANs, and the corresponding spectrum demonstrated the true signals coming from the 2D-band of GIANs (Fig. 3e). When the 2D-band was used for bioimaging, the endogenous species did not generate any interference. Thus, bioimaging with the 2D-band of GAIN avoided background interference and provided much more accurate imaging results.

### Multiplexed SERS imaging with isotopic GIANs *in vitro* and *in vivo*

**Fig. 4:**
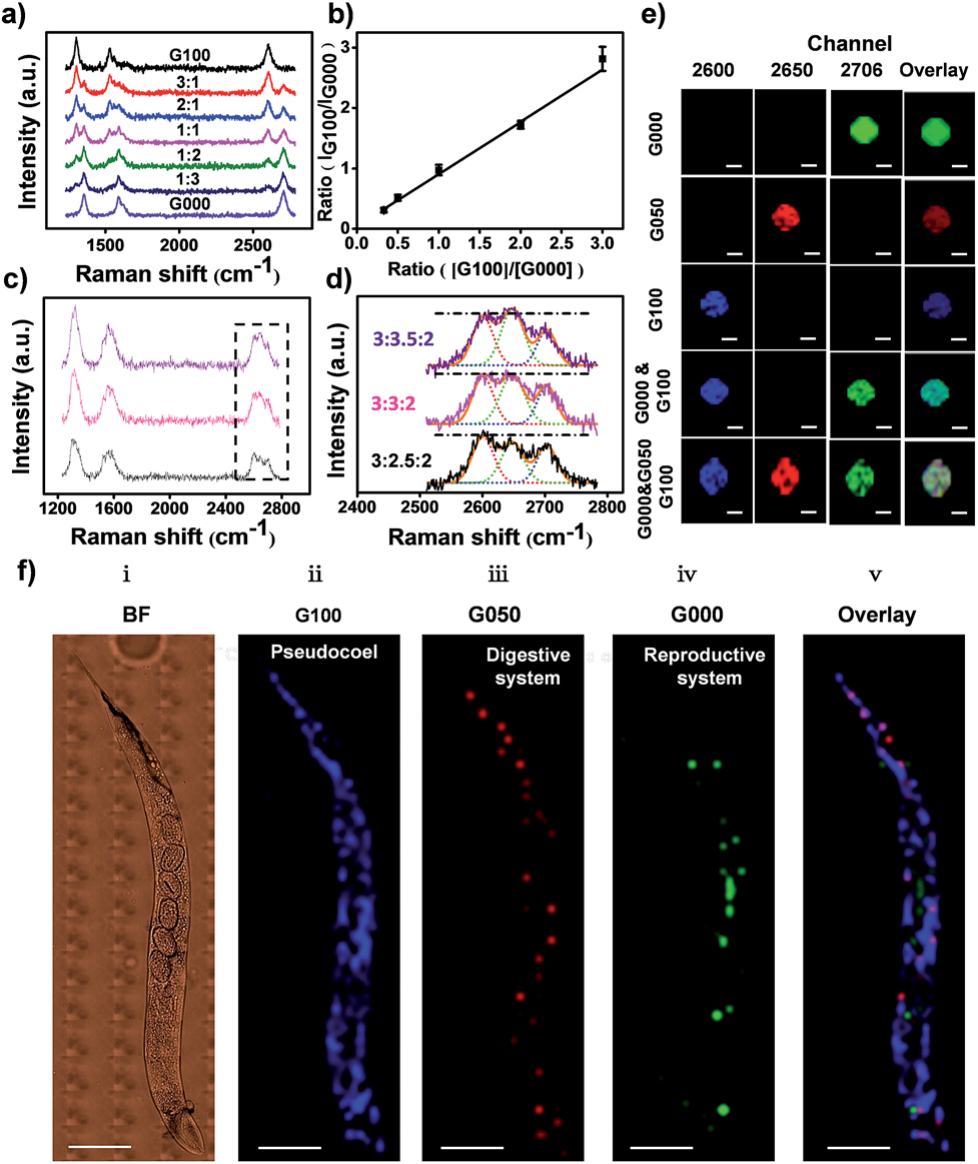
Multiplexed SERS imaging with isotopic GIANs *in vitro* and *in vivo*. (a) SERS spectra of mixed G100 and G000, from top to bottom, in varying ratios (*C*_G100_ : *C*_G000_) of 3 : 1, 2 : 1, 1 : 1, 1 : 2, and 1 : 3. (b) Linear fitting of (a). (c) SERS spectra of mixed G100, G050 and G000 and fixed concentration of G100 and G000, while decreasing the concentration of G050, as G100 : G050 : G000 of 3 : 2.5 : 2, 3 : 3 : 2 and 3 : 3.5 : 2. (d) Gaussian fitting of enlarged spectra of 2D-bands corresponding to boxed area in (c), 2D-band fitting of G100 (blue dotted line), G050 (red dotted line) and G000 (green dotted line). (e) SERS mapping images acquired in 2D-band of G100 (blue, 2600 cm^–1^), G050 (red, 2650 cm^–1^), G000 (green, 2706 cm^–1^) and overlay of images. GIAN mixtures were at equal concentrations; scale bar, 10 μm. (f) (i) Bright-field (BF) of *C. elegans*. (ii–v) SERS imaging of *C. elegans* with 2D-band of (ii) G100 (blue), pseudocoel; (iii) G050 (red), digestive system; (iv) G000 (green), reproductive system; (v) overlay of G100, G050 and G000. Step size, 11 μm, scale bar, 200 μm.

With superior Raman imaging quality, three isotopic GIAN tags (G100, G050, and G000) were utilized to demonstrate multiplexed SERS imaging capability. We investigated the resolution of Raman analysis by mixing G100 and G000 at different ratios (Fig. 4a and b). The 2D-band of these two mixed tags could be distinguished completely, as shown in Fig. 4a. The ratios of 2D-band intensity (*I*_G100_/*I*_G000_) showed a good linear relationship with the ratios of GIAN concentration (*C*_G100_/*C*_G000_) (Fig. 4b). Fig. 4c showed SERS spectra of G100, G050 and G000 mixtures at different ratios. We fixed the concentrations of G100 and G000 and changed the concentration of G050. As the concentration of G050 decreased, the 2D-band intensity of G050 also decreased. Since the Raman shifts of 2D-band were larger than those of the D-band and G-band, the 2D-band showed higher multiplexed spectrum resolution compared to either D- or G-band. The enlarged spectra of 2D-bands corresponding to the boxed area in Fig. 4c were fitted by Gaussian algorithm (Fig. 4d). Fig. 4e illustrated the mapping of multiplexed SERS imaging with isotopic GIAN tags in capillary. Multiplexed SERS mapping of dual-color images was conducted with mixtures of G100 and G000 (Fig. 4e, fourth row). Signals of two channels corresponding to G100 (blue) and G000 (green) were obtained. Furthermore, multiplexed SERS mapping of tri-color images were conducted with mixtures of G100, G050 and G000 (Fig. 4e, last row). Signals of three channels corresponding to G100 (blue), G050 (red) and G000 (green) were obtained. All these results demonstrated multiplexed SERS imaging capability *in vitro*.

We further demonstrated the feasibility of isotopic cellular Raman-silent GIANs-based multiplexed bioimaging *in vivo* by again using *C. elegans* as the model.^65^ The pseudocoel, digestive and reproductive system of *C. elegans* were microinjected with G100, G050 and G000 tags, respectively, followed by observation through confocal Raman microspectroscopy. No background interference was observed at the Raman imaging region of *C. elegans*, as indicated in Fig. S8.† The 2D-band SERS signal of G100 (blue) was clearly visible in the pseudocoel of *C. elegans*, while the SERS 2D-band signal of G050 (red) was detected in the digestive system of *C. elegans*. The reproductive system was illuminated by the G000 tag (green) (Fig. 4f). The organs of *C. elegans* were visualized simultaneously through multiplexed isotopic GIAN tags. The isotopic GIAN tags presented superior multiplexed SERS imaging capabilities in this living system using rather simple instrumentation, a result which shows promise for more *in vivo* applications.

### Cancer cell pattern recognition with aptamer-functionalized isotopic GIAN tags

**Fig. 5:**
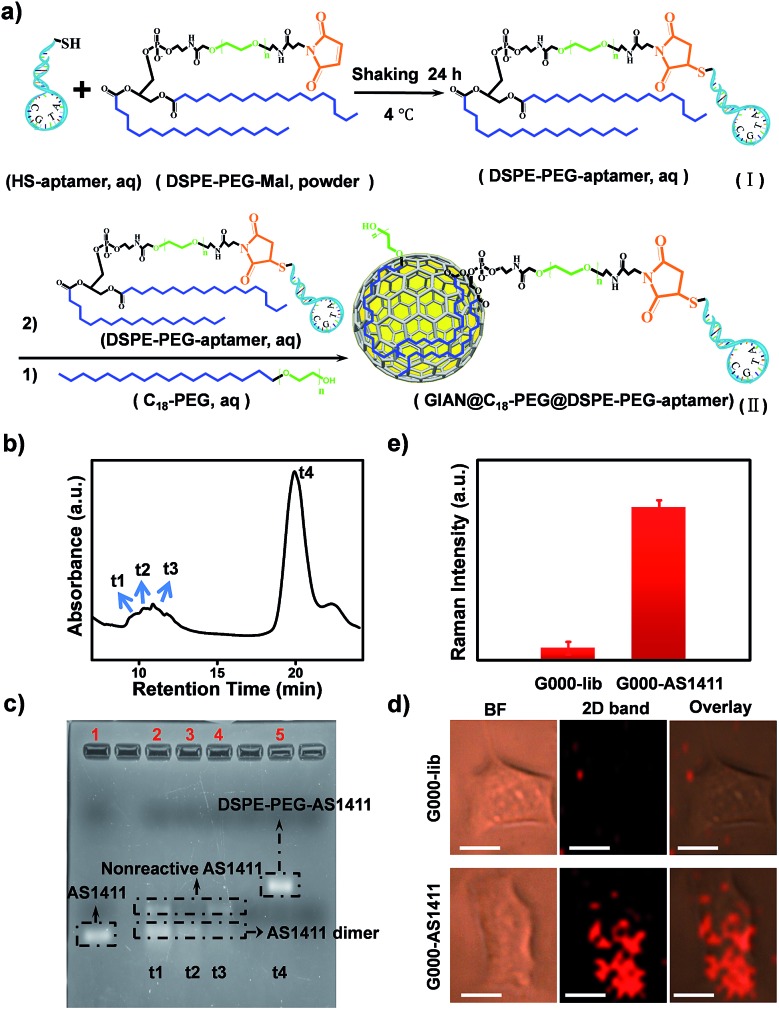
Targeting strategy for cancer cells based on GIAN-aptamer complexes. (a) Schematic illustration of aptamer-functionalized GIAN. (b) DSPE-PEG-AS1411 purified by reverse phase HPLC. (c) Agarose gel electrophoresis characterized the DSPE-PEG-linked aptamers (DSPE-PEG-AS1411) separated by HPLC. (d) SERS imaging of A549 cancer cells incubated with G000-lib (top) and G000-AS1411 (bottom); scale bar, 10 μm. (e) Statistics of 2D-band SERS intensity are shown in (d); the error bar came from three independent experiments.

SERS-encoding technologies have been widely used for multiplexed biosensing, dramatically improving analytical efficiency and accuracy. Compared to traditional Raman reporters, isotopic GIAN tags presented a very simple and stable fingerprint spectrum and avoided the interference between SERS-encoders. Meanwhile, GIAN tags were demonstrated to have low interference from background biomolecules. With superior multiplexed SERS imaging capabilities in complex biosystems, isotopic GIANs were utilized for cancer cell recognition. Surface functionalization was first performed to enable the specificity of the isotopic GIAN-encoders. Aptamers, which can be chemically modified and synthesized by automatic DNA synthesizer, are cost-effective chemical antibodies that have specific affinity to proteins located on the cancer cell membrane. Modifying isotopic GIANs with different aptamers could confer them with the ability for multiplexed cancer cell discrimination. Fig. 5a illustrated the strategy for fabrication of GIAN-aptamer-encoders. Thiol-labeled aptamer was cross-linked with phospholipid-polyethyleneglycol-maleimide (DSPE-PEG3400-Mal) (Fig. 5a(i)). The alkyl DSPE chain helped anchor the aptamer to the graphitic GIANs surface through hydrophobic–hydrophobic interaction (Fig. 5a(ii)).^38^ Meanwhile, the PEGlinker with flexible long chain as the bridge between the aptamer and NP could greatly enhance the freedom of aptamer to improve cell recognition.^66^ The synthesized DSPE-PEG-aptamers were purified and collected with reversed-phase high performance liquid chromatography (HPLC). In Fig. 5b, the phospholipid-polyethylene glycol-linked aptamer AS1411 (DSPE-PEG-AS1411) targeting nucleolin overexpressed on the cancer cell surface was separated from reaction mixture through HPLC. The larger polar molecules showed shorter retention time. DSPE-PEG-AS1411 possessed the smallest polar molecules and presented longest retention time at *t*_4_, while the nonreactive AS1411 and its dimer showed shorter retention time (*t*_1_, *t*_2_ and *t*_3_). Agarose gel electrophoresis was further utilized to characterize DSPE-PEG-AS1411 separated by HPLC. As in the gel image (Fig. 5c), DSPE-PEG-AS1411 (lane 5, corresponding to peak *t*_4_ in Fig. 5b) migrated much slower than pure AS1411 monomer (lane 1) and nonreactive AS1411 aptamers (lane 2, lane 3 and lane 4, corresponding to peak *t*_1_, *t*_2_ and *t*_3_ in Fig. 5b). By changing the base sequence, we obtained the other two DSPE-PEG-linked aptamers, DSPE-PEG-S1.6 binding MUC1 and DSPE-PEG-SYL3C targeting EpCAM (Fig. S9a†). As illustrated in the UV-Vis spectra (Fig. S9b†), DSPE-PEG-AS1411 aptamers were successfully conjugated to the GIAN. Through this strategy, three GIAN-encoders were obtained.

**Fig. 6:**
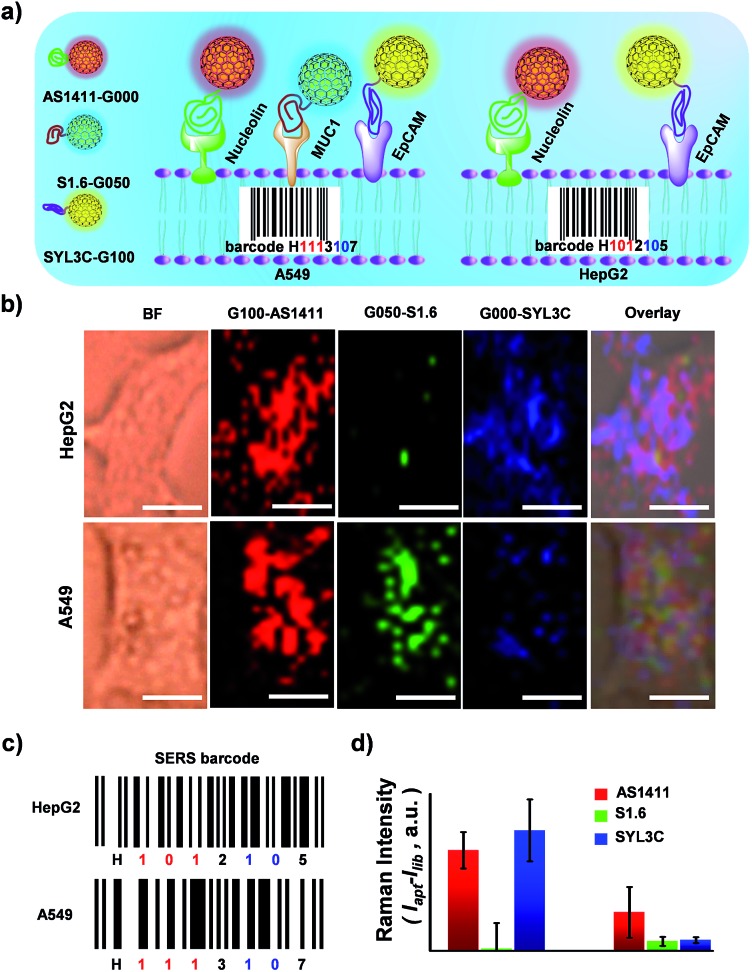
Cancer cell pattern recognition with aptamer-functionalized isotopic GIAN-encoders. (a) Schematic illustration of pattern recognition and discrimination of cancer cell lines with multiplexed GIAN-encoders. (b) SERS images of cancer cells, scale bar, 10 μm. G100, G050 and G000 conjugated with DSPE-PEG-linked aptamer AS1411, S1.6 and SYL3C, respectively. (c) SERS barcodes of HepG2 and A549 cell lines. (d) Statistics of normalized SERS signals shown in (b).

To demonstrate the nonspecific absorption resistance capability of the aptamer-functionalized GIANs for cancer cell recognition, control molecule was synthesized by replacing the AS1411 aptamer with a library sequence (lib) with no targeting ability. DSPE-PEG-AS1411 and DSPE-PEG-lib were conjugated to G000 individually and then incubated with a non-small cell lung cancer cell line, A549, which is known to overexpress nucleolin on the cell membrane, respectively. The A549 cells were recognized by G000-AS1411. Fig. 5d showed the SERS mapping images of A549 cells. The cells incubated with G000-AS1411 showed strong SERS signal from the G000 tags, while cells incubated with G000-lib had almost no signals. Statistical analysis of SERS images from three independent experiments were demonstrated in Fig. 5e. More SERS mapping images of A549 cells with G000-AS1411 were shown in the Fig. S10,† and all indicated cancer cell targeted recognition capability of the G000-AS1411 complexes. The recognition capability of G000-SYL3C and G000-S1.6 were also demonstrated as shown in Fig. S11.† All these results indicated superior targeting and nonspecific absorption resistance capability of the DSPE-PEG-aptamer-functionalized GIANs, showing their potential for more biomedical applications. Imaging technologies must possess both inherent molecular recognition and built-in pattern recognition for efficient target cancer cell discrimination.^35^ With their superior targeting and nonspecific absorption resistance capabilities, these unique isotopic GIAN-aptamer SERS-encoders were next applied for pattern recognition of two cancer cell lines. As schematically illustrated in Fig. 6a, non-small cell lung cancer cell line A549 and liver cancer cell line HepG2 could be efficiently distinguished through pattern recognition with these isotopic GIAN-encoders. Nucleolin, MUC1 and EpCAM proteins were overexpressed on the membrane of the A549 cell line. Meanwhile, only two of these three proteins, nucleolin and EpCAM, were overexpressed on the surface of the HepG2 cell line, thus distinguishing it from the A549 cell line.^67^,^68^ Nucleolin, MUC1 and EpCAM proteins can be targeted by aptamer AS1411, S1.6 and SYL3C, respectively.^51^–^54^ G100, G050 and G000 were individually conjugated with the DSPE-PEG-linked aptamers AS1411, S1.6 and SYL3C. These three isotopic GIAN SERS-encoders were incubated with each cancer cell line together in the same cell dish for 2.5 hours, followed by SERS mapping. Library sequence severed as the control and control experiments were performed with cells incubated with G100-lib, G050-lib and G000-lib. As shown in Fig. S12,† cells were not stained by these negative GIANs. In Fig. 6b, images in the top row were the SERS mapping images of HepG2 cell line, and SERS mapping images of A549 cell line were in the bottom row. The HepG2 cell line was decorated with two GIAN-encoders, G100-AS411 and G000-SYL3C, while the A549 cell line was decorated with three encoders, G100-AS1411, G050-S1.6 and G000-SYL3C. Two unique patterns of molecular recognition were obtained, and the results of molecular recognition were consistent with conditions of proteins overexpressed on these two cell lines. We defined the positive SERS signal of cancer cell as “1”, while the negative signal was defined as “0” and human cancer cell as “H”. Code values of isotopic GIAN SERS signals were added together and the value defined as the fifth code. Furthermore,“10” was defined as adherent cells. Finally, all numbers of each cell line were added together and defined as last code of each barcode. Thus, the HepG2 cell line had SERS barcode “H1012105”, and the A549 cell line had barcode “H1113107” (Fig. 6c). According to the SERS barcode, A549 cells and HepG2 cells could be discriminated efficiently. Fluorescence flow cytometry was further utilized to confirm aptamer recognition (Fig. S13†). A549 cells were targeted by three aptamers, while HepG2 cells were targeted by AS1411 and SYL3C. The binding patterns of aptamers were consistent with SERS-encoded images, while the GIAN-encoding required only one laser, less time and exhibited less background interference. Statistical values for SERS imaging were shown in Fig. 6d. These unique GIAN-encoders showed promise for efficient targeted cancer cell imaging and discrimination.

## Conclusions

We fabricated multiplexed SERS tags with simple and low background fingerprint spectra with isotopic cellular Raman-silent GIANs. The 2D-band of GIAN was regulated very easily by CVD of isotopic carbon sources. Five GIAN tags with simple, but super-stable, optical properties were synthesized. The Stokes shift of 2D-band between G000 and G100 was 106 cm^–1^, about 1.9 and 1.8 times greater than the shift of D-band and G-band, respectively. Dark-field microscopy imaging on few isotopic GIANs demonstrated high sensitivity and precise Raman shift. Multiplexed sensitive SERS imaging was demonstrated without background interference from the biomolecules *in vitro* and *in vivo*. Moreover, isotopic GIANs were functionalized with synthesized phospholipid-polyethylene glycol-linked aptamers and targeted cancer cells. SERS-encoding with GIANs was used for multiplexed bioimaging, dramatically improving analytical efficiency and accuracy. G100-AS1411, G050-S1.6 and G000-SYL3C GIAN SERS-encoders were developed for pattern recognition of HepG2 and A549 cell lines. Two different SERS barcodes were obtained. The results of molecular pattern recognition were consistent with conditions of proteins overexpressed on these two cell lines. We realized efficient cancer cell discrimination through an isotopic GIAN SERS-encoding strategy. GIAN-aptamer-encoders showed high potential for cost-effective cancer cell identification with high sensitivity and low background interference, promising a novel tool for more biomedical applications.

## Author contributions

Z. C., W. T. and Y. Z. conceived the project, designed the experiments and wrote the manuscript. Y. Z. conducted all the experiments. S. H., H. T. and Y. L. assisted with *C. elegans* experiments. X. Z. and Y. C. assisted with FDTD simulation and optical scattering experiments. L. C. and X. H. contributed to data analysis and cell culture. F. L., Z. L. and L. Z. assisted with TEM characterization. All authors discussed the results and commented on the manuscript.

## Conflicts of interest

There are no conflicts to declare.

## Supplementary Material

Supplementary information
