# Supplementary material for: Isotopic graphene–isolated-Au-nanocrystals with cellular Raman-silent signals for cancer cell pattern recognition
Source: Chem Sci. 2018 Feb 12;9(10):2842–9. doi: 10.1039/c7sc05442d (PMC5914539; doi:10.1039/c7sc05442d)
Supplement: Supplementary file 1 [file SC-009-C7SC05442D-s001.pdf]

Electronic Supplementary Material (ESI) for Chemical Science.
This journal is © The Royal Society of Chemistry 2018

Electronic Supplementary Information

Isotopic graphene-isolated-Au-nanocrystals with cellular Raman-silent signals for cancer cell pattern recognition

Yuxiu Zou,[a] Siqi Huang,[a] Yixin Liao,[a] Xupeng Zhu,[b] Yiqin Chen,[b] Long Chen,[c] Fang Liu,[a] Xiaoxiao Hu,[a] Haijun Tu,[a] Liang Zhang,[a] Zhangkun Liu,[a] Zhuo Chen[a,]* and Weihong Tan[a,d]

[a]Molecular Science and Biomedicine Laboratory (MBL), State Key Laboratory of Chemo/Bio-Sensing and Chemometrics, College of Chemistry and Chemical Engineering and College of Life Sciences, Aptamer Engineering Center of Hunan Province, Hunan University, Changsha Hunan, 410082(China). **E-mail: zhuochen@hnu.edu.cn*

[b]State Key Laboratory of Advanced Design and Manufacturing for Vehicle Body, College of Mechanical and Vehicle Engineering, Hunan University, Changsha Hunan, 410082(China).

[c]Faculty of Science and Technology, University of Macau, E11, Avenida da Universidade, Taipa, 999078, Macau.

[d]Department of Chemistry and Department of Physiology and Functional Genomics, Center for Research at Bio/nano Interface, Health Cancer Center, UF Genetics Institute and McKnight Brain Institute, University of Florida, Gainesville, Florida 32611-7200 (USA).

Experimental Procedures **S3**

Supporting Figures **S6**

Figure S1. Optical stability and optimization of GIANs S6

Figure S2. DLS data of GIANs before and after functionalized with C_{18}-PEG S6

Figure S3. Images of GIANs incubated harsh and complex solutions S6

Figure S4. UV-Vis spectra of GIANs incubated harsh and complex solutions S7

Figure S5. Quantitative detection of GIANs S7

Figure S6. FDTD simulation of GIAN S8

Figure S7. Raman characterization of multiplexed isotopic GIANs S8

Figure S8. SERS images of untreated *C. elegans*. S8

Figure S9. Functionalization of GIANs S9

Figure S10. SERS mapping images of large area. S9

Figure S11. SERS mapping images of A549 cells targeted by G000-SYL3C and G000-S1.6 .... S9

Figure S12. SERS mapping images of HepG2 and A549 cells incubated with G100-lib, G050-lib and G000-lib S10

Figure S13. Targeting demonstration with flow cytometry S10

References **S10**

Experimental Procedures

Reagents and Materials. $HAuCl_4 \cdot 4H_2O$ and Biowest agarose were purchased from Sinopharm Chemical Reagent Company (China); Hydrophilic Silicon dioxide were from Aladdin; C13 methane was bought from Newradar Special Gas Company (China). Polyoxyethylene stearyl ether (C_{18}-PEG, MW 4670), tris(2-carboxyethyl) phosphine hydrochloride (TCEP), ethidium bromide (EB) and acetonitrile were obtained from Sigma Aldrich. 0.1 M trimethylamine acetate (TEAA) was purchased from Glen Research Corp. Thiol-marked aptamers were bought from Sangon Biotech (China). Phospholipid-polyethylene glycol-maleimide (DSPE-PEG-Mal, MW 3400) was purchased from Laysan Bio. RPMI 1640 medium, DMEM medium, and dulbecco's phosphate buffered solution (dPBS) were bought from Gibco. Fetal bovine serum was bought from Thermo Fisher Scientific. 10×TBE buffer (108 mg tris base, 55 mg boric acid, 9.3 mg Na_4 EDTA, 1 liter H_2O) and ultrapure water (resistance >18 MΩ cm^{-1}) was used throughout all experiments. *C. elegans* (wild type, N2 Bristol), human lung adenocarcinoma cell line (A549), human embryonic kidney 293 cell line (HEK293) and liver cancer cell line (HepG2) were bought from ATCC.

Table S1. Detailed sequence information for all oligonucleotide probes

DNA	5' to 3'
HS-Lib	HS-AAAAAAAAAATTTTTTAATTATTTATATTAAT
HS-AS1411	HS-AAAAAAAAGGTGGTGGTGGTTGTGGTGGTGGTGG
HS-S1.6	HS-GGGAGACAAGAATAAACGCTCAAGCAGTTGATCCTTTGGATACCCTGGTTCGACAGGAGGCTCACAACAGGC
HS-SYL3C	HS-AAAACACTACAGAGGTTGCGTCTGTCCCACGTTGTCATGGGGGGTTGGCCTG
Lib-FITC	AAAAAAAAAATTTTTTAATTATTTATATTAAT-FITC
AS1411-FITC	AAAAAAAAGGTGGTGGTGGTTGTGGTGGTGGTGG-FITC
S1.6-FITC	GGGAGACAAGAATAAACGCTCAAGCAGTTGATCCTTTGGATACCCTGGTTCGACAGGAGGCTCACAACAGGC-FITC
SYL3C-FITC	AAAACACTACAGAGGTTGCGTCTGTCCCACGTTGTCATGGGGGGTTGGCCTG-FITC

Characterization. Transmission electron microscopic (TEM) images were collected using a JEOL 3010, operated at 120 to 200 kV. The DLS data of GIAN was obtained from Malvern Zeta sizer Nano ZS90. Raman measurements were performed on confocal laser Raman microspectroscopy (Renishaw's InVia Raman System) and the spectral resolution is 1 cm^{-1}. SERS spectra of several NPs performed on confocal laser Raman microspectroscopy (WITec-300R, Germany), with dark field microscopy (100× magnification , NA=0.9, ZEISS, Germany). UV-Vis spectrum was obtained from UV-Vis spectrophotometer (UV-2450, Shimadzu Corporation, Japan). Cross-linked aptamers were purified by HPLC (Agilent 1260, USA) with C_{18} reversed phase column (Inertsil ODS-3, GL Sciences Inc., Japan). Flow cytometry (BD Accuri cytometer) were utilized to characterize the binding ability of aptamer. *C. elegans* was microinjected with nanoparticles through microinjection system (Eppendorf InjectMan® 4; Eppendorf, Germany).

The finite-difference time-domain (FDTD) simulation. FDTD solution 8.0 (Lumerical Solutions Inc.) was used to calculate the near-field distribution. The size of both GIANs was determined by TEM. The complex dielectric constant of gold and cobalt was from the database of Johnson-Christy and http://www.filmetrics.com/, respectively. To simulate the electric field distribution under the excitation of laser focused by objective lens, total scattering power source was chosen as the light source. The mesh size was automatically defined according to the 8th precision level, and a fine mesh was added for all models with X, Y, Z direction of 0.2 nm. We set a perfectly matched layer (PML) boundary condition for all three dimensions. The source frequency at 532 nm, 633 nm and 785 nm was used to simulate laser excitation.

Synthesis of GIANs and isotopic GIANs. The synthesis of isotopic GIANs was performed on a silica-supported gold catalyst using methane CVD growth following our previously reported protocol at 1000 °C. Briefly, fumed silica (1.00 g) was impregnated with 6 mL $HAuCl_4·4H_2O$ methanol solution (1%) in 160 mL methanol and sonicated for 1 h, and then eliminated the solution and dried at 65 °C. Typically, 0.50 g of the powder was used for methane CVD in a tube furnace. The sample grew with a methane flow of 150 $cm^3·min^{-1}$ for 8 min. To control isotope compositions of GIANs, C12 and C13 methane gas were mixed at desired ratios by flow controllers during the growth of GIANs. The as-grown product was etched in 15% HF solution in water at room temperature to remove the silica support. The purified GIANs were then washed thoroughly with distilled water to neutral pH and stored at 4 °C for further use. To achieve better solubility, a polyoxyethylene stearyl ether (C_{18}-PEG) molecule was introduced to functionalize GIANs through hydrophobic-hydrophobic interaction. Consequently, GIANs were attached polyoxyethylene stearyl ether molecule and adequately dispersed in water.

The vibrational frequency depends on the atomic mass and the spring force constant following Equation I:

$$\nu = \frac{1}{2\pi c}\sqrt{\frac{k}{\mu}} \quad (I)$$

Equation 1

Where ν is the wavenumber of the vibration (in cm^{-1}), c is the speed of light (ms^{-1}), k is the force constant of a diatomic bond (Nm^{-1}), and μ is the reduced mass (kg) given by Equation II:

$$\mu = \frac{m_1 \cdot m_2}{m_1 + m_2} \quad (II)$$

Equation 2

Where m_i are the masses of both atoms. By substituting atoms with the heavier analogues, a red-shift of the corresponding Raman band is the consequence.

In a continuum model, the frequency shift of the Raman bands in the C13 enriched GIAN material originates from the increased mass of this isotope which is given by equation:

$$\nu = \nu_0 \sqrt{(12 + n_0)/(12 + n_{13})} \quad [III]$$

Equation 3

where ν_0 is the frequency of a particular Raman mode in the C12 sample, n_{13} = 0.99 is the fraction of C13 in the enriched sample, and n_0 = 0.0107 is the natural abundance of C13.[1, 2]

SERS on few isotopic GIAN nanoparticles. C_{18}-PEG functionalized GIANs suspension (46 pM) was dropped on silicon substrate and dried at room. Under the leading of dark field microscopy imaging, we obtained SERS signals of multiplexed GIAN from few NPs with 100× magnification, 10 s integration time and 532 nm laser (18 μW/μm^2).

***C. elegans* Maintenance, Microinjection and SERS imaging.** *C. elegans* of wild-type N2 Bristol was maintained on *Escherichia coli* OP50 seeded nematode growth media (NGM) plates at 20 °C. To multiplexed label different parts of *C. elegans,* G100, G050 and G000 were microinjected into pseudocoel, digestive system and reproductive system of the same worm individually, and then the injected worms were examined with confocal Raman microspectroscopy. The microinjection was performed by using the Eppendorf InjectMan® 4. In parallel, the analysis was also performed on the untreated worm as the control. Pseudocoel of *C. elegans* was microinjected with G000s. And then characterized by Raman confocal microscopy with 11 μm step size, 50× magnification, 1 s integration time per pixel.

Functionalization of GIAN. Thiol-marked aptamers were conjugated with DSPE-PEG-Mal. First, aptamers were mixed with 5 mM TCEP overnight at room temperature, adjusting pH to 7. After that, DSPE-PEG-Mal powder (34

mg) was added to aptamers (0.6 mL, 10 μM) (~ 1.7:1 molar ratio) and shaken at 4 °C overnight. Finally, the crosslinked production was purified by HPLC using 0.1 M TEAA and acetonitrile as the eluent. The products of DSPE-PEG-linked aptamers were characterized by agarose gel electrophoresis gel (2%, 110 voltage, running time 40 minutes).Through change the DNA sequence, other cross-linked DNA molecules were synthesized. Then 10 μL DSPE-PEG-aptamers (6 μM) were mixed with 50 μL GIANs suspension (2 nM). After 4 hour incubation, free DSPE-PEG aptamers were eliminated. The alkyl DSPE chain helped anchor the aptamer to the graphitic GIANs surface, while PEG linker with flexible long chain as the bridge between the aptamer and NP could greatly enhance the freedom of aptamer to improve cell recognition.

Cell culture. A549 cells were cultured at 37 °C in RPMI 1640 medium supplemented with 10% premium fetal bovine serum (FBS) and a 5% CO_2 environment. HepG2 and HEK293 cells were cultured at 37 °C in DMEM medium supplemented with 10% premium fetal bovine serum (FBS) and a 5% CO_2 environment.

Cell uptake of GIAN-encoders and SERS imaging. Cancer cells were incubated with GIANs-aptamer complexes at 37°C in dPBS for 2.5 hours. For SERS imaging, the cell dish was washed three times with dPBS. And then characterized by Raman confocal microscopy with 1.2 μm step size, 50× magnification, 1 s integration time per pixel.

Characterization of binding ability through flow cytometry methods. A549, HepG2 and HEK293 cells were cultivated on cell dish two days. The cells were washed three times with dPBS. Then, cells individually incubated with AS1411-FITC, S1.6-FITC, SYL3C-FITC and Lib-FITC at 37 °C for 30 minutes, followed by washing three times with dPBS. All the concentration of aptamer was 250 nM. Then, the binding ability was determined by flow cytometry.

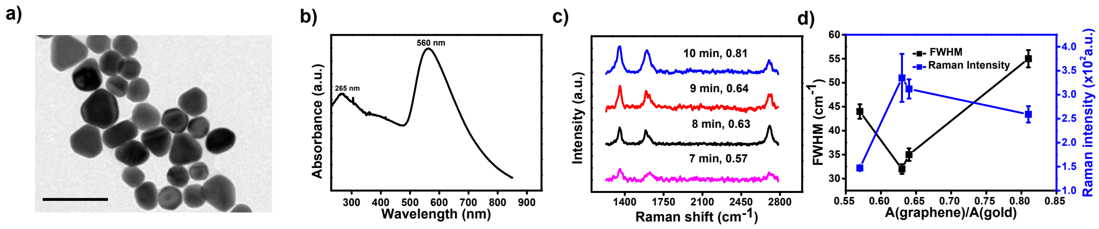

Figure S1. Optical stability and optimization of GIANs. (a) TEM of GIANs; scale bar, 100 nm. (b) UV-Vis spectrum of aqueous GIANs. (c) The Raman spectra of GIANs at different growth time. (d) FWHM and Raman intensity of 2D-band with increased ratios of A_{265}/A_{560}, including 0.57, 0.63, 0.64, 0.81, whose time on CVD of methane was 7, 8, 9, 10 minutes, respectively.

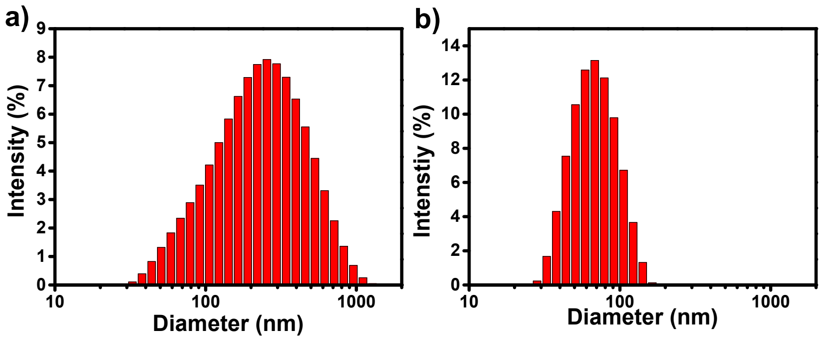

Figure S2. DLS data of GIANs before (a) and after (b) functionalized with C18-PEG. After the surface modification, GIANs were well dispersed in water and demonstrated superior stability.

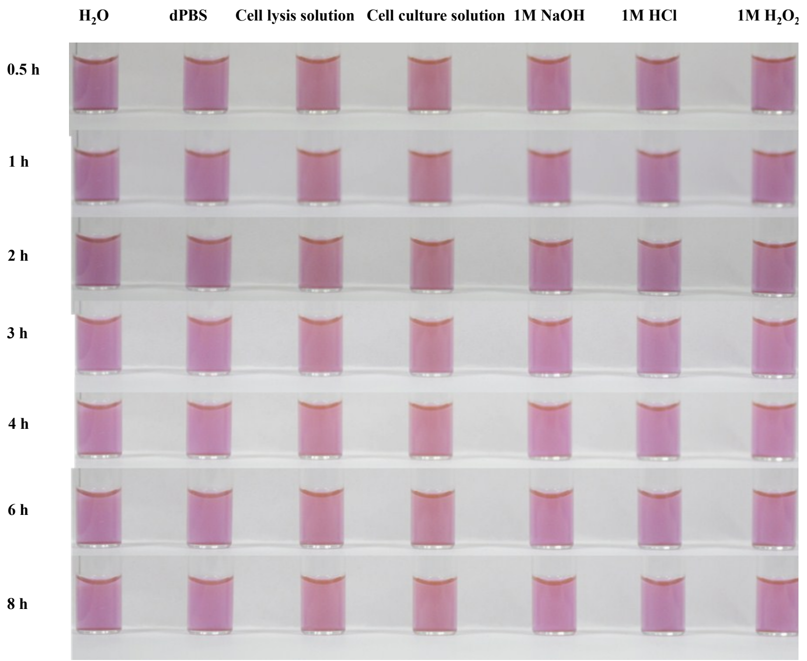

Figure S3. Images of GIANs incubated with H_2O, dPBS, cell lysis solution, cell culture solution, 1 M NaOH, 1 M HCl and 1 M H_2O_2 at various times.

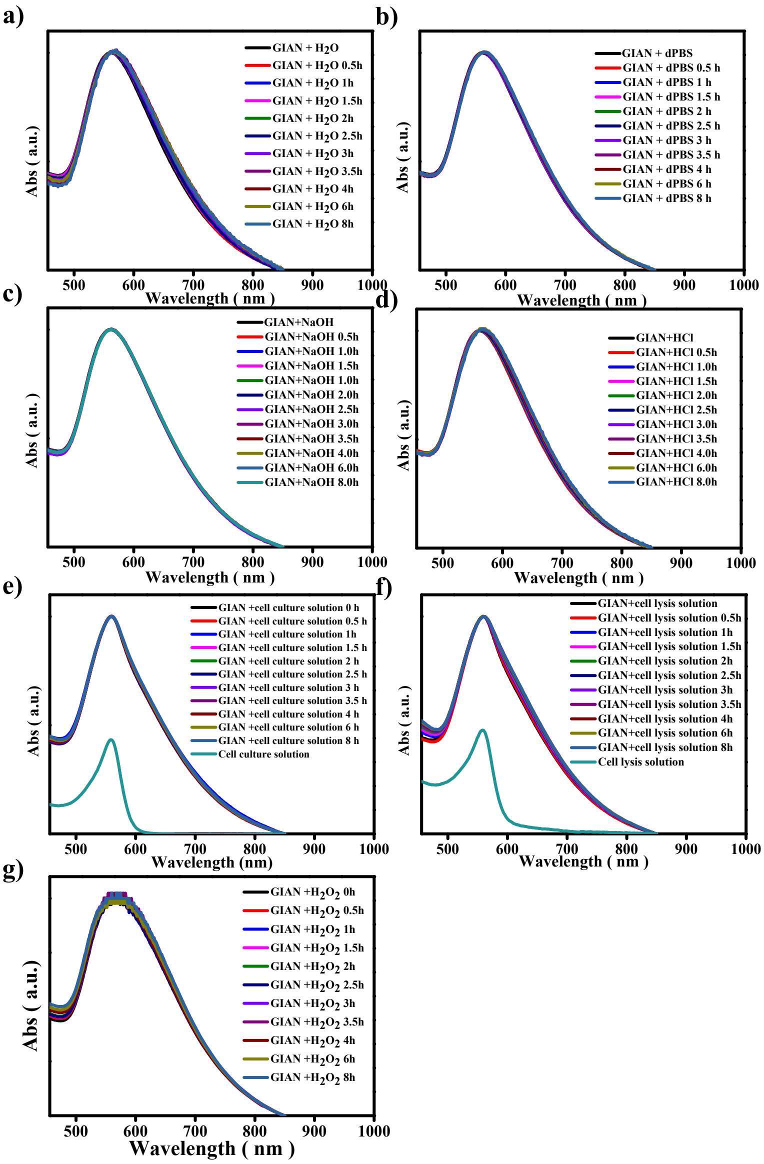

Figure S4. UV-Vis spectra of GIANs suspension. (a), (b), (c), (d), (e), (f), and (g) UV-Vis spectra of GIANs mixtures incubated with H_2O, dPBS, 1 M NaOH, 1 M HCl, cell culture solution, cell lysis solution and 1 M H_2O_2, respectively. The baby blue line represents the UV-Vis spectra of cell culture solution (e) and cell lysis solution.

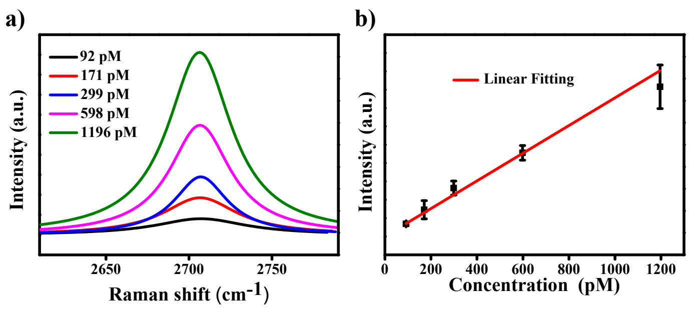

Figure S5. Quantitative detection of GIANs. (a) SERS spectra of GIANs suspension with various concentrations, under 20 second integrating time. (b) The scatter diagram and linear fitting of (a).

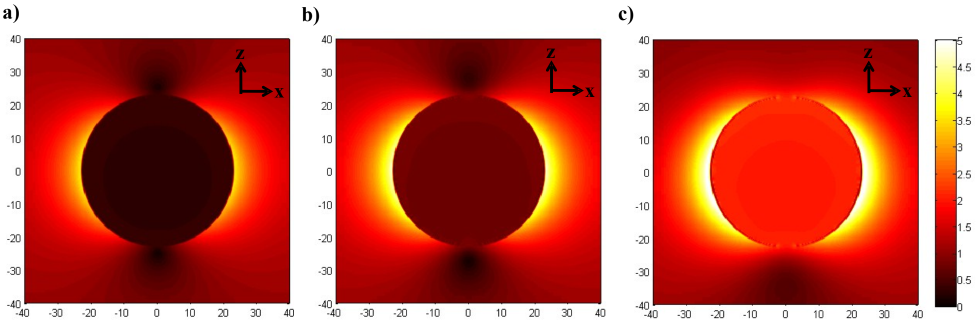

Figure S6. FDTD simulation of GIAN with 45 nm average diameter. (a) Electric field distribution under 785 nm laser. (b) Electric field distribution under 633 nm laser. (c) Electric field distribution under 532 nm laser.

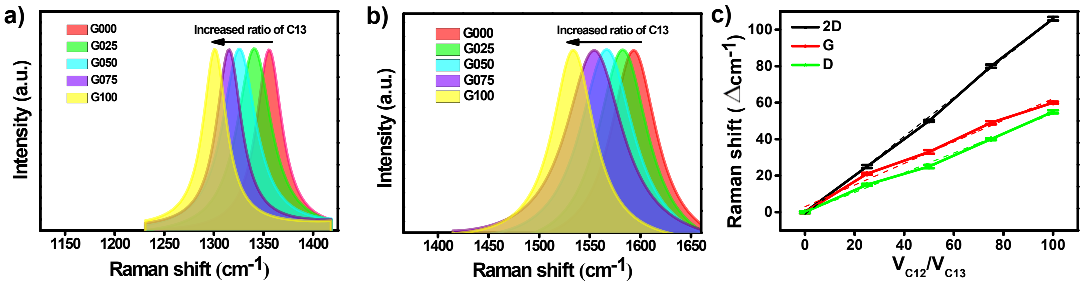

Figure S7. Raman characterization of multiplexed isotopic GIANs. (a) Raman spectra of D-band and (b) G-band of GIANs with different fractions of C13 methane. From right to left, the ratios of C13 methane were 0%, 25%, 50%, 75%, and 100%. (c) The plot line and linear fitting relationship between the fraction of C13 methane and the value of Raman shifts. Error bar depended on 10 spectra. Results showed that he shift of 2D-band at about twice the frequency of D-band. The larger isotopic shift for the 2D mode is because the shift is relative to the frequency of the particular mode.

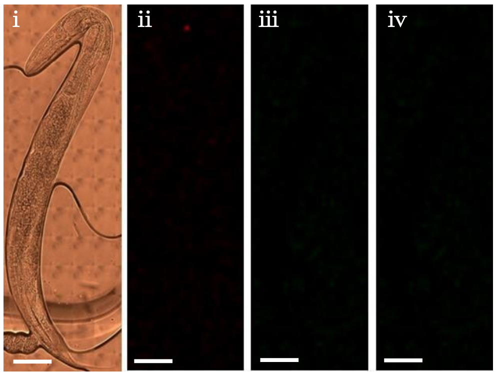

Figure S8. SERS images of untreated *C. elegans*. (i) Bright-field; SERS images of *C. elegans* acquired with (ii) 2600 cm^{-1} , (iii) 2650 cm^{-1} and (iv) 2706 cm^{-1}. Scale bar, 200 μm.

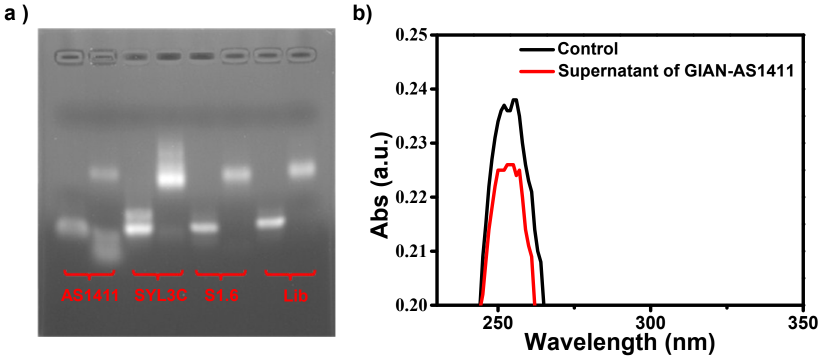

Figure S9. (a) Gel electrophoresis characterized the DSPE-PEG-linked aptamers, AS1411, SYL3C, S1.6 and lib sequence (lib) separated by HPLC system. (b) UV-Vis spectra of supernatant of GIANs mixed with DSPE-PEG-AS1411.

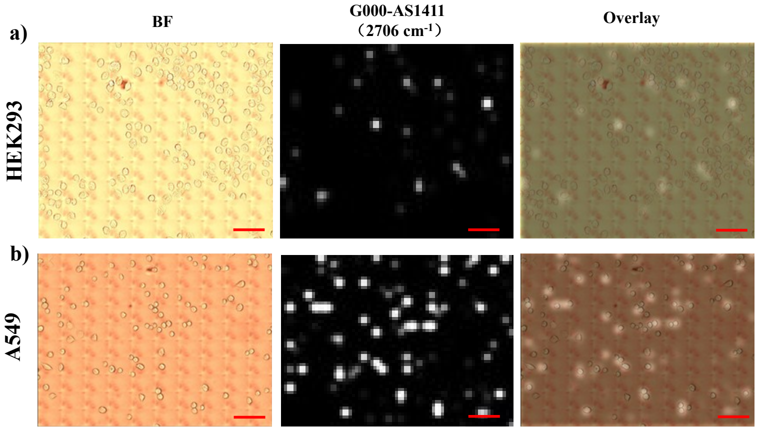

Figure S10. SERS mapping images of large area. (a) Human embryonic kidney 293 cells (HEK293) and (b) A549 cells cultured with G000-AS1411. Step size, 11 µm; scale bar, 100 µm; 50 × magnification. HEK 293 was the negative cell line.

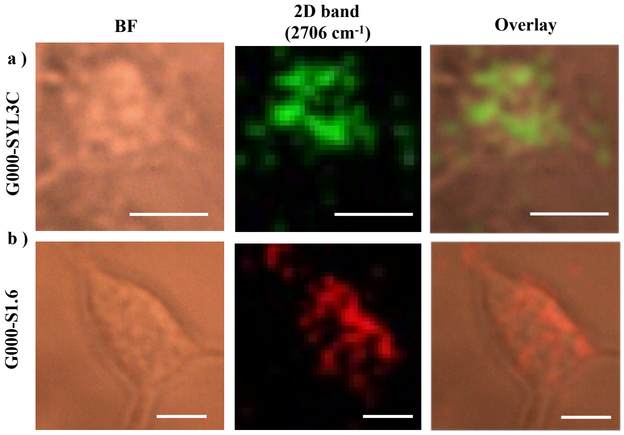

Figure S11. SERS mapping images of A549 cells targeted with (a) G000-SYL3C (b) and G000-S1.6, individually. Scale bar, 10 µm.

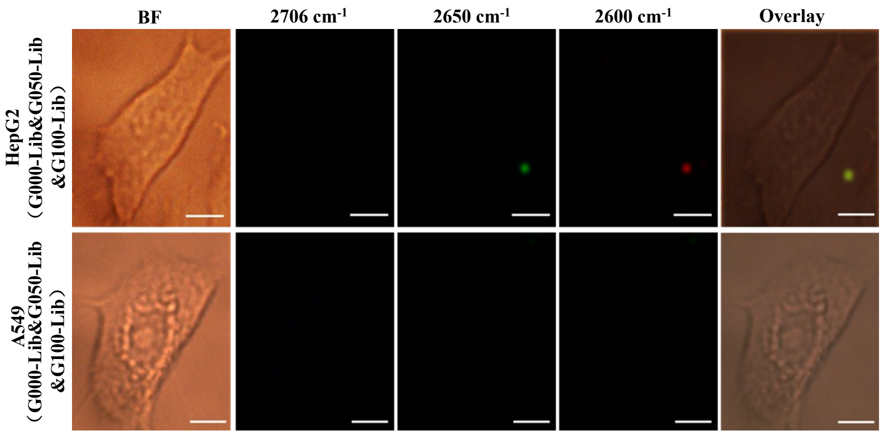

Figure S12. SERS mapping images of HepG2 cells (upper row) and A549 cells (lower row) incubated with G100-lib, G050-lib and G000-lib composites; scale bar, 10 μm.

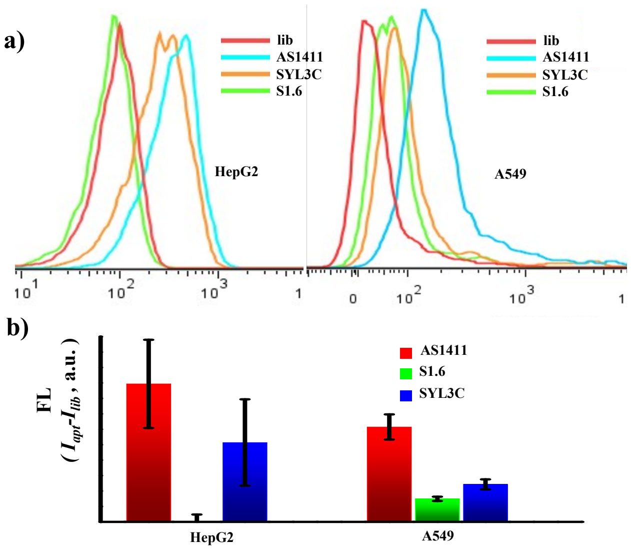

Figure S13. Targeting demonstration with flow cytometry. (a) Flow cytometry of aptamers binding HepG2 and A549 cell lines individually. (b) Fluorescence of flow cytometry in (a).

References

1. M. Kalbac, H. Farhat, J. Kong, P. Janda, L. Kavan and M. S. Dresselhaus, *Nano Lett.*, 2011, **11**, 1957-1963.
2. F. Shoushan, L. Liang and L. Ming, *Nanotechnology*, 2003, **14**, 1118.
